# Carcinoid Syndrome: A Review

**DOI:** 10.7759/cureus.7186

**Published:** 2020-03-05

**Authors:** Ajay K Gade, Eva Olariu, Nathan T Douthit

**Affiliations:** 1 Internal Medicine, Brookwood Baptist Medical Center, Birmingham, USA; 2 Internal Medicine, Brookwood Baptist Health, Birmingham, USA; 3 Medical Education, Brookwood Baptist Health, Birmingham, USA

**Keywords:** carcinoid syndrome, carcinoid, 5-ht

## Abstract

Carcinoid syndrome (CS) is a paraneoplastic syndrome caused by the release of serotonin and other substances from well-differentiated neuroendocrine tumors (NETs). The hallmark symptoms of carcinoid syndrome are flushing and diarrhea; atypical signs and symptoms can include wheezing, abdominal pain, valvular heart disease, telangiectasias, pellagra, and the complications of mesenteric fibrosis, including ureteral obstruction, bowel obstruction, and bowel ischemia. These symptoms are mediated by the release of serotonin (5-HT), histamine, kallikrein, prostaglandins, and tachykinins. The diagnosis of CS requires these symptoms and corresponding elevations in lab tests. Treatment options include surgery and medical management with somatostatin analogs.

## Introduction and background

Carcinoid syndrome (CS) is a paraneoplastic syndrome caused by the release of serotonin and other substances from well-differentiated neuroendocrine tumors (NETs) [1]. The hallmark symptoms of carcinoid syndrome are flushing and diarrhea; atypical signs and symptoms can include wheezing, abdominal pain, valvular heart disease, telangiectasias, pellagra, and the complications of mesenteric fibrosis, including ureteral obstruction, bowel obstruction, and bowel ischemia [1]. These symptoms are mediated by the release of serotonin (5-HT), histamine, kallikrein, prostaglandins, and tachykinins [2]. The diagnosis of CS requires these symptoms and corresponding elevations in lab tests. Treatment options include surgery and medical management with somatostatin analogs representing the cornerstone of therapy.

## Review

Epidemiology

The incidence of NETs is 2.7 per 100,000 population, whereas the incidence of the carcinoid syndrome is 0.27 per 100,000 population in the United States [3]. CS affects men and women equally, with African-Americans affected more than the other ethnic groups [3].

Pathophysiology

CS is a paraneoplastic syndrome associated with the secretion of approximately 40 vasoactive hormones, predominantly 5-hydroxytryptamine (5-HT) [3]. CS also involves the secretion of histamine (primary gastric NETs), kallikrein, prostaglandins E and F, and tachykinins. NETs can arise in the foregut, midgut, or hindgut (Table 1) [1-3].

**Table 1:** Gastrointestinal tract

	Foregut	Midgut	Hindgut
Begins	Esophagus	3rd part of the duodenum	Distal transverse colon
Ends	2nd part of the duodenum	Proximal transverse colon	Anus

Tumors may also arise from the bronchi, gonads, or thymus. CS is most commonly caused by NETs of the midgut. The foregut tumors lack the aromatic amino acid decarboxylase that converts 5-hydroxytryptophan (5-HTP) to 5-HT; these tumors produce 5-HTP and histamine instead of 5-HT and rarely produce some of the symptoms of CS [4]. Hindgut tumors from the distal colon and rectum rarely secrete 5-HT or any other vasoactive hormones and are, therefore, unassociated with hormonal syndromes even when metastatic [5]. In a patient with normal hepatic function, the 5-HT and kallikrein are metabolized by the liver and the manifestations of carcinoid syndrome do not occur unless liver metastasis occurs [1]. If the intestinal NET metastasizes to the liver, these chemicals cannot be metabolized releasing the metabolic products via the hepatic veins directly into the systemic circulation [6]. CS may also be the result of liver failure or cirrhosis. However, there are a few exceptions such as ovarian or lung carcinoid, when the venous blood from a NET enters directly into the systemic circulation. The excessive 5-HT finally undergoes an oxidative reaction in the presence of aldehyde dehydrogenase to form 5-hydroxy indole acetic acid (5-HIAA) [7]. 5-HIAA is renally excreted.

5-HT increases peristalsis of the gut, limiting the time for the fluid absorption and eventually leading to watery diarrhea [8]. Prostaglandins also mediate increased intestinal motility and fluid secretion in the gastrointestinal tract causing diarrhea [9]. Skin flushing results from 5-HT as well as kallikrein, which catalyzes the conversion of kininogen to lysyl-bradykinin, which, in turn, is converted to bradykinin, a strong vasodilator [10]. In a patient with NET, up to 70% of tryptophan is converted into 5-HT, as opposed to approximately 1% in a normal patient. This leads to the diversion of the large amounts of the tryptophan from the synthesis of the niacin, eventually causing pellagra (dermatitis/diarrhea/dementia) [11]. 5-HT may also stimulate fibroblast growth and fibrogenesis. These effects can lead to retroperitoneal and mesenteric fibrosis as well as cardiac valvular fibrosis. Complications of mesenteric fibrosis may include intra-abdominal vessel ischemia and intestinal obstruction as well as ureteral obstruction and renal failure [1]. CS may cause fibrotic lesions of the endocardium, particularly on the right side of the heart, resulting in an insufficiency of the tricuspid valve and, less frequently, the pulmonary valve [12-13]. In many patients, the cause of death is attributed directly to cardiac disease. 5-HT is inactivated in the lungs so left-heart involvement is rare and may indicate an intra-atrial shunt [14-15]. Uncommonly, CS may cause bronchoconstriction. The exact pathogenesis of the cardiac lesions and bronchoconstriction is unknown [1]. 

**Figure 1:**
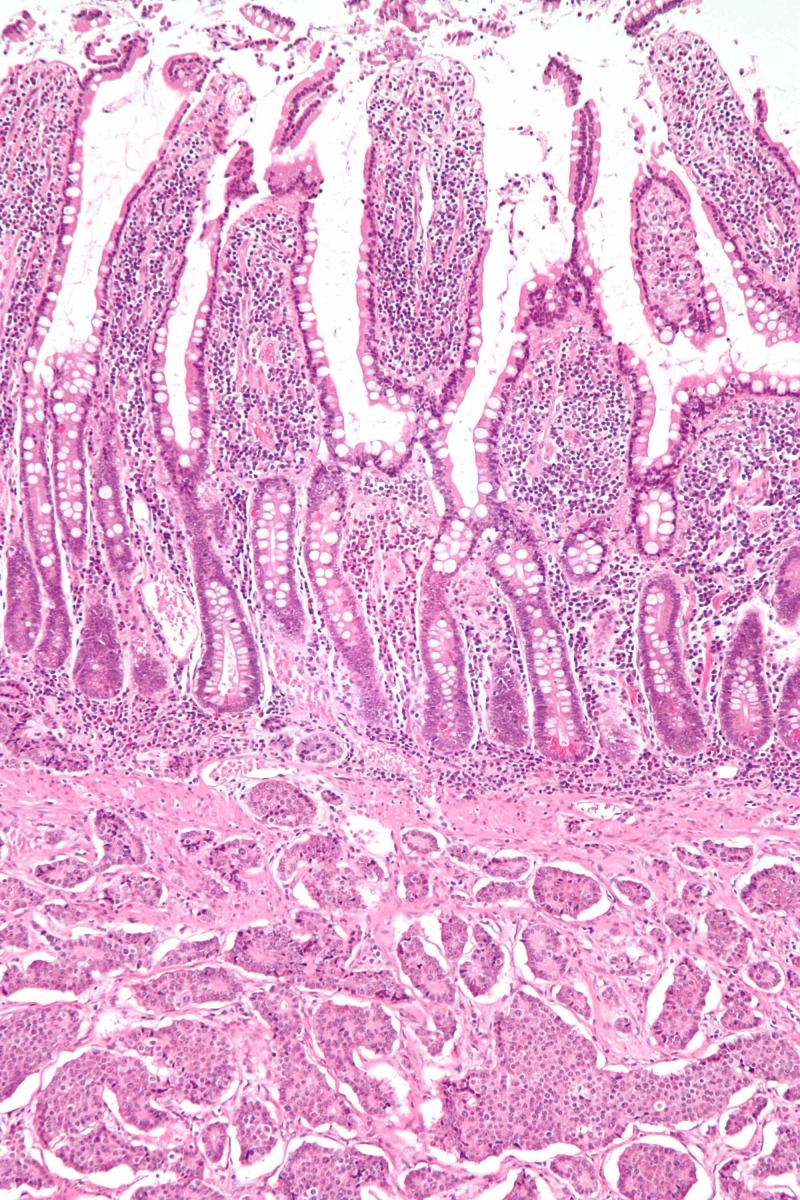
Histopathology of a neuroendocrine tumor of the small bowel (H&E stain) The submucosal tumor that infiltrates the muscularis propria's trabecular architecture, with solid nests of cells with fibrous stroma in between; moderate, finely granular cytoplasm, small nucleoli, and stippled chromatin or salt-and-pepper chromatin. (*Image credit: Librepathology)* H&E: hematoxylin and eosin

A carcinoid crisis is a potentially life-threatening complication of carcinoid syndrome caused by the sudden release of 5-HT and other vasoactive peptides, such as histamine, kallikreins, or catecholamines, which are precipitated by tumor manipulation during surgery, percutaneous needle biopsy, or even anesthesia [1,16]. It manifests as hypotension or hypertension, diarrhea, bronchoconstriction, flushing, and acidosis [17]. Figure 1 shows the histopathology of a neuroendocrine tumor of the small bowel.

Diagnosis

Clinical

CS is associated with several common symptoms. Classically, patients complain of flushing (90%), diarrhea (70%), and wheezing (15%) [1,18]. The flushing is usually sudden-onset, pink or red in color, and involves the face or trunk. It lasts only a few minutes and can appear many times in a day. It may be triggered by alcohol, exercise, or tyramine-containing foods (chocolate, walnuts, bananas) [19]. It is not associated with sweating. The flushing may present atypically with a purplish color that lasts several hours and may involve the limbs. Finally, a bright red patchy flush may also present, likely induced by histamine [7]. Diarrhea is frequently described as secretory. It is generally intermittent in the beginning but may become continuous if untreated. Wheezing is generally secondary to bronchoconstriction, most likely secondary to tachykinins and bradykinins. Valvular heart disease is common, although rarely clinically significant on presentation. These patients should be screened for any murmurs on the cardiac exam. Chronic diarrhea and excessive tryptophan synthesis can lead to niacin depletion and pellagra [20]. A carcinoid crisis is a life-threatening complication of carcinoid, generally associated with sedatives or anesthesia but can be a presenting factor as well in rare cases. It is characterized by severe flushing, diarrhea, hypotension, and arrhythmias. Prophylactic continuous intravenous octreotide infusion is usually given during the procedures to prevent a carcinoid crisis [20-21].

Laboratory

Urinary 5-hydroxyindoleacetic acid: 24-hour urine 5-HIAA is the initial laboratory test of choice. Serotonin released by carcinoid tumors is metabolized by monoamine oxidases in the liver, lungs, and brain to 5-HIAA [22]. When measured in a 24-hour urine sample, 5-HIAA level has a sensitivity of 73% and a specificity of 100% for diagnosing carcinoid [22]. Urine 5-HIAA can also be detected in people who eat food rich in tryptophan, to prevent these false-positive results one should abstain from tryptophan-containing foods 72 hrs before the test [23].

Chromogranin concentration: Chromogranin (Cg) is a nonspecific tumor marker for neuroendocrine tumors. There are three types of chromogranins: CgA, CgB, and CgC. Chromogranin A is commonly used due to its high sensitivity [24]. CgA cannot be used for screening, as it has low specificity, but it can be used to assess disease progression, response to therapy, or recurrence after surgical resection [25].

Plasma 5-HIAA: Plasma 5-HIAA concentration is a relatively more convenient test for the patients as opposed to the 24 hr urinary 5-HIAA [26]. The measurement of both serum and plasma 5-hydroxyindoleacetic acid can be used for the diagnosis and monitoring of patients with neuroendocrine tumors [27]. Provided renal function is taken into consideration, either of these tests should be incorporated into standard practice as an alternative assay to urinary 5-hydroxyindoleacetic acid [28].

Other laboratory tests: Plasma serotonin concentration and urinary excretion of serotonin are very less commonly used biochemical tests [29]. Blood/ urine serotonin levels are not recommended as a standard diagnostic test as the end product of the serotonin metabolism is 5-HIAA [30].

Imaging

X-ray: There are two instances where X-rays are helpful. A bronchial carcinoid can be seen on chest X-ray and is characterized by round or oval opacities with sharp and notched margins with associated hilar or perihilar mass [31]. It is occasionally associated with airway compression with pulmonary atelectasis. The other instance where X-rays are helpful is in the setting of a thymic carcinoid tumor, which is characterized by focal areas of necrosis or punctate calcifications on chest X-ray [31].

**Figure 2:**
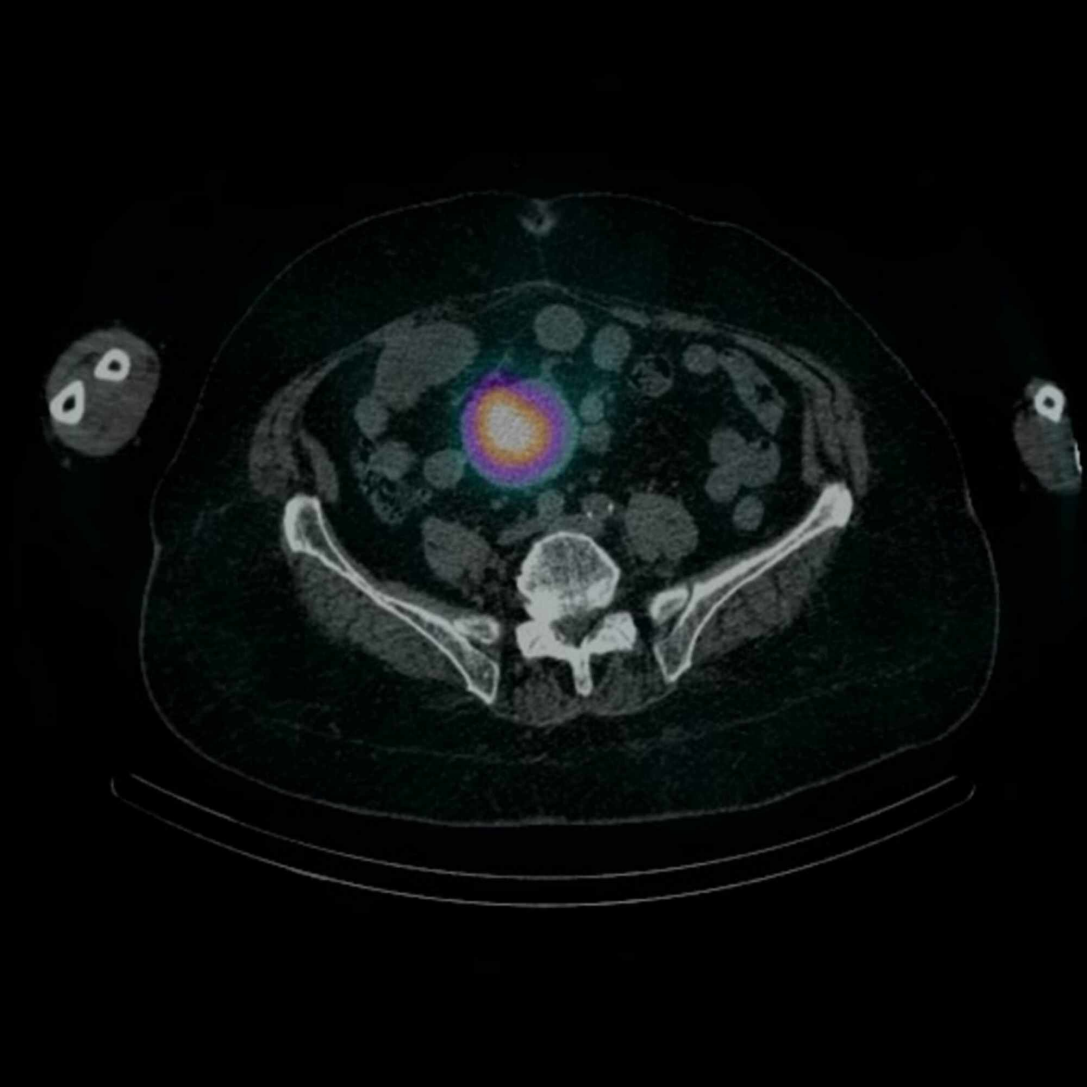
Indium-111 octreotide concentrates in the gastrointestinal tract carcinoid tumors as well as in liver metastases. Case courtesy of Dr. Henry Knipe, Radiopaedia.org, rID: 42873

Somatostatin receptor scintigraphy: Somatostatin scintigraphy may detect resectable tumors that would be unrecognized with regular imaging studies [32]. The radionucleotides used in this test are indium-111 octreotide and indium-111 pentetreotide. The degree of uptake is directly proportional to the density of the receptors. The radionucleotides used in this test are indium-111 octreotide (Figure 2) and indium-111 pentetreotide. Somatostatin receptor scintigraphy alone or in combination with other imaging modalities led to the detection of more tumor sites than any combination of imaging studies [33].

Echocardiography (TTE): TTE should be performed in all patients with carcinoid syndrome and a high suspicion for cardiac involvement consistent with clinical features such as cardiac murmurs, raised brain natriuretic peptide (BNP), or pro-b-type natriuretic peptide (proBNP) with or without thickening and retraction of immobile tricuspid valve leaflets and pulmonary valve cusps with associated tricuspid regurgitation [34]. Transesophageal echocardiography provides a better assessment of the degree of valvular involvement and the atrial septal anatomy.

Other diagnostic imaging: Computed tomography (CT) is of poor utility in detecting primary carcinoid tumors but is helpful in evaluating the extent of tumor spread before surgical exploration. An abdominal magnetic resonance imaging (MRI) scan may be performed to detect metastases of carcinoid syndrome to the liver and mesentery [35]. Cardiac MRI can be used to identify cardiac metastases. Capsule endoscopy and double-balloon enteroscopy are also useful diagnostic tools for detecting primary carcinoids. More studies and research is required to clarify their potential complications and relationship with other techniques, particularly nuclear imaging [36-37].

Staging

The American Joint Committee on Cancer (AJCC) stages carcinoid syndrome as shown in Tables 2-4 [38].

**Table 2:** Carcinoid tumors of the jejunum or ileum

American Joint Committee on Cancer (AJCC) stage	Staging group and description
I	T1- the tumor is no more than 1 cm across and has grown from the top layer of cells and into deeper layers, such as the lamina propria or submucosa, N0- No lymph nodal involvement, and M0- No Metastasis.
II	T2- the tumor has grown into the lamina propria or submucosa (or both) and is greater than 1 cm across, OR the tumor has grown into the muscularis propria, N0- No lymph nodal involvement, M0- No Metastasis. OR T3- the tumor has grown through the muscularis propria and into the subserosa, N0- No lymph nodal involvement, and M0- No Metastasis.
III	T4- the tumor has grown into the serosa or visceral peritoneum or into nearby organs or structures, N0- No lymph nodal involvement, and M0- No Metastasis OR Any T- tumor can be any size and might or might not have grown into nearby structures, N1 or N2- It has spread to nearby lymph nodes, and M0-No Metastasis.
IV	Any T- tumor can be any size and might or might not have grown into nearby structures, Any N- It might or might not have spread to nearby lymph nodes, and M1- cancer has spread to distant parts of the body.

**Table 3:** Carcinoid tumors of the stomach

American Joint Committee on Cancer (AJCC) stage	Staging group and description
I	T1- the tumor is no more than 1 cm across and has grown from the top layer of cells and into deeper layers, such as the lamina propria or submucosa, N0- cancer has not spread to nearby lymph nodes, and M0- cancer has not spread to distant parts of the body.
II	T2- the tumor has grown into the lamina propria or submucosa (or both) and is greater than 1 cm across, OR the tumor has grown into the main muscle layer of the stomach (the muscularis propria), N0- cancer has not spread to nearby lymph nodes, and M0- cancer has not spread to distant parts of the body.
III	T4- the tumor has grown into the outer layer of tissue covering the stomach (the serosa or visceral peritoneum) or into nearby organs or structures. N0- cancer has not spread to nearby lymph nodes, M0- cancer has not spread to distant parts of the body, OR Any T- tumor can be any size and might or might not have grown into nearby structures, N1- It has spread to nearby lymph nodes, and M0- Cancer has not spread to distant parts of the body.
IV	Any T- tumor can be any size and might or might not have grown into nearby structures, Any N- It might or might not have spread to nearby lymph nodes, and M1- cancer has spread to distant parts of the body.

**Table 4:** Carcinoid tumors of the colon or rectum

I	T1- the tumor is no more than 2 cm across and has grown from the top layer of cells and into deeper layers, such as the lamina propria or submucosa, N0- cancer has not spread to nearby lymph nodes, and M0- cancer has not spread to distant parts of the body.
IIA	T2- the tumor has grown into the lamina propria or submucosa (or both) and is greater than 2 cm across, OR the tumor has grown into the muscularis propria, N0- Cancer has not spread to nearby lymph nodes, and M0- Cancer has not spread to distant parts of the body.
IIB	T3- the tumor has grown through the muscularis propria and into the subserosa, N0- cancer has not spread to nearby lymph nodes, and M0- cancer has not spread to distant parts of the body.
IIIA	T4- the tumor has grown into the outer layer of tissue covering the intestine (the serosa or visceral peritoneum) or into nearby organs or structures, N0- cancer has not spread to nearby lymph nodes, and M0- cancer has not spread to distant parts of the body.
IIIB	Any T- tumor can be any size and might or might not have grown into nearby structures, N1- It has spread to nearby lymph nodes, and M0- tumor has not spread to distant parts of the body.
IV	Any T- tumor can be any size and might or might not have grown into nearby structures, Any N- It might or might not have spread to nearby lymph nodes, and M1- cancer has spread to distant parts of the body.

Treatment / Management

Medical Management

Somatostatin analogs: Somatostatin analogs (Octreotide, Sandostatin, Lanreotide) may reduce the symptoms of carcinoid syndrome, including skin flushing and diarrhea [39]. Somatostatin analogs are synthetic forms of somatostatin, a pancreatic hormone that acts by binding to somatostatin receptors expressed on the majority of carcinoid tumors. Approximately 80% of carcinoid tumors express somatostatin receptors [40]. Octreotide and Lanreotide have also been proven to inhibit tumor growth in randomized phase III trials [41]. A depot form of octreotide, which is administered intramuscularly on a monthly basis, as well as a long-acting formulation of lanreotide, is available for monthly injections [42]. Somatostatin analogs also inhibit gallbladder contractility, which can lead to gallstones or sludge. Prophylactic cholecystectomy is occasionally recommended for patients who are undergoing abdominal surgery for other reasons. They can be used to prevent or treat carcinoid crises before, during, and after procedures such as surgery and embolization [43]. Antidiarrheals/anticholinergics, such as Loperamide and diphenoxylate-atropine, are used for diarrhea. Serotonin receptor antagonists, such as Ondansetron, can also be used. Pancreatic malabsorption may be a contributing factor, which can be alleviated with pancreatic enzyme supplementation.

Telotristat: Tryptophan is converted to serotonin in the presence of the enzyme tryptophan hydroxylase. An oral tryptophan hydroxylase inhibitor, telotristat has recently been approved in the United States for use in combination with somatostatin analog therapy in order to control diarrhea associated with carcinoid syndrome [44].

Interferon: Another option for the control of refractory symptoms of the carcinoid syndrome in patients treated with somatostatin analogs is interferon alfa (IFNa). Interferons can exert antitumor effects via the stimulation of T cells, induction of cell cycle arrest, and inhibition of angiogenesis [45].

Chemotherapy

Chemotherapy drugs may shrink neuroendocrine tumors, particularly those of pancreatic origin. These include temozolomide, fluorouracil, oxaliplatin, everolimus, and streptozotocin-based chemotherapy regimens. Chemotherapy is generally not helpful for the control of symptoms in patients with carcinoid syndrome from well-differentiated carcinoid tumors. Its use should be restricted primarily to patients with high-grade/poorly differentiated neuroendocrine tumors [46].

Cytotoxic treatment is the first-line treatment for malignant neuroendocrine tumors in the pancreas and for gastric carcinoids if the Ki67 antibody level is greater than 10%. It may be a second-line treatment if other means of treatment fail [19].

Surgical Management

Cytoreductive surgery is the mainstay in the surgical treatment of carcinoid syndrome. Surgery is reserved for patients in whom 90% of the tumor bulk can be removed in the absence of diffuse bi-lobar involvement, compromised liver function, or widespread extrahepatic metastases. Hepatic resection can control the symptoms of carcinoid syndrome [47]. Carcinoid crisis is a serious complication during hepatic resection or biopsy due to tumor manipulation. This can be prevented with the pre and intraoperative administration of Octreotide. For airway lesions, surgeries such as lobectomy, sleeve resection, or pneumonectomy may be performed depending on the size and location of the tumor [17].

Locoregional Therapies

Embolization is a therapy to treat liver tumors by blocking their blood supply. Because liver tumors thrive on highly oxygenated blood from the hepatic artery, blocking that supply may kill it. Embolization of the entire liver can be undertaken for patients with bilobar disease. Bland embolization uses only microparticles whereas chemoembolization, also known as transarterial chemoembolization (TACE), uses chemotherapy in addition to microparticles. Patients undergoing HAE should also receive pre and post embolization octreotide to prevent a carcinoid crisis [48].

Radiofrequency ablation delivers heat through a needle to the metastatic cells in the liver, causing cell death. Microwave ablation destroys liver tumors using the heat generated by microwave energy. Radioembolization using yttrium-90 (90Y)-labeled resin or glass microspheres is growing [49].

Prognosis 

Prognosis of carcinoid tumors is generally good and the five-year survival rate of patients is approximately 75.1% in the stomach, 76.1% in the small intestine, 76.1% in the appendix, and 87.5% in the rectum [50]. The factors that influence the prognosis are the site of origin within the gastrointestinal tract (GI) tract, the size of the primary tumor, and the anatomical extent of the disease, whether localized, regional, or metastatic to distant sites. The bad prognostic factors are carcinoid heart disease and high levels of tumor markers, liver metastasis, the involvement of the thymus, overexpression of the proliferation of Ki67, and mutation of the p53 gene [5].

## Conclusions

Carcinoid syndrome is a rare paraneoplastic syndrome caused by the discharge of serotonin and other vasoactive substances from well-differentiated neuroendocrine tumors. In an affected individual with ordinary hepatic function, 5-HT and kallikrein are metabolized through the liver and the manifestations of carcinoid syndrome do not occur until liver metastasis occurs. Complications from this ailment include retroperitoneal fibrosis, cardiac disease, and carcinoid crisis. The diagnosis is made based on scientific and laboratory findings with ancillary roles for imaging. Treatment is primarily based on somatostatin analogs, however, there is a role for surgical procedures and different locoregional therapies. The disease has a relatively good prognosis if recognized early in its course and managed appropriately.
